# IRE1α-XBP1 inhibitors exerted anti-tumor activities in Ewing’s sarcoma

**DOI:** 10.18632/oncotarget.24467

**Published:** 2018-02-12

**Authors:** Yu Tanabe, Yoshiyuki Suehara, Shinji Kohsaka, Takuo Hayashi, Keisuke Akaike, Kenta Mukaihara, Taisei Kurihara, Youngji Kim, Taketo Okubo, Midori Ishii, Saiko Kazuno, Kazuo Kaneko, Tsuyoshi Saito

**Affiliations:** ^1^ Department of Orthopedic Surgery, Juntendo University School of Medicine, Bunkyo-ku, Tokyo 113-8421, Japan; ^2^ Department of Medical Genomics, Graduate School of Medicine, The University of Tokyo, Tokyo 113-0033, Japan; ^3^ Department of Human Pathology, Juntendo University School of Medicine, Bunkyo-ku, Tokyo 113-8421, Japan; ^4^ Laboratory of Proteomics and Biomolecular Science, Research Support Center, Juntendo University School of Medicine, Bunkyo-ku, Tokyo 113-8421, Japan

**Keywords:** Ewing's sarcoma (ES), proteomics, EWS/FLI1, XBP1, unfolded protein response (UPR)

## Abstract

Ewing's sarcoma (ES) is the second-most frequent pediatric bone tumor. Chromosomal translocation t(11;22)(q24:q12) results in the formation of EWS/FLI1 gene fusion, which is detected in approximately 90% of tumors of the Ewing family. Several transcriptome studies have provided lists of genes associated with EWS/FLI1 expression. However, the protein expression profiles associated with EWS/FLI1 have yet to be elucidated. In this study, to identify the regulated proteins associated with EWS/FLI1 and therapeutic targets in ES, we conducted proteomic studies using EWS/FLI1 knockdown in four Ewing's sarcoma cell lines and human mesenchymal stem cells (hMSCs) expressing EWS/FLI1. Isobaric tags for relative and absolute quantitation (i-TRAQ) analyses identified more than 2,000 proteins regulated by the EWS/FLI1 fusion. In addition, the network analyses identified several critical pathways, including XBP1, which was ranked the highest. XBP1 is a protein well known to play an important role in the unfolded protein response (UPR) to endoplasmic reticulum (ER) stress through the IRE1α-XBP1 pathway. We confirmed the high mRNA expression of XBP1 (spliced XBP1 and unspliced XBPl) in surgical samples and cell lines in ES. The silencing of XBP1 significantly suppressed the cell viabilities in ES cell lines. In the inhibitor assays using IRE1α-XBP1 inhibitors, including toyocamycin, we confirmed that these agents significantly suppressed the cell viabilities, leading to apoptosis in ES cells both *in vitro* and *in vivo*. Our findings suggested that IRE1α-XBP1 inhibitors might be useful for developing novel therapeutic strategies in ES.

## INTRODUCTION

Ewing sarcoma (ES) is a malignant tumor of bone and soft tissue and the second-most common primary bone tumor, more frequently affecting young adults than other age groups [1]. Clinically, ES is highly aggressive with a high propensity for relapse and metastasis [1–4]. Genetically, ES is characterized by the presence of EWS-FLI1 or other related gene fusions. Chromosomal translocation t(11;22)(q24:q12) results in the formation of EWS/FLI1 gene fusion, which is detected in approximately 90% of tumors of the ES family, and recent studies suggest that ES may arise from the malignant transformation of mesenchymal and/or neural crest stem cells [1].

However, despite significant progress regarding intensive chemotherapy and improvements in the outcomes of patients with localized ES, relapse after initial clinical remission is not uncommon, and the overall survival for patients with relapsed or metastatic ES remains less than 20% [1, 3–8]. Therefore, the development of novel therapeutic targets that inhibit biological pathways known to contribute to ES growth and malignant behavior is essential.

Recent comprehensive studies have offered a global view of the molecular aberrations associated with the malignant spectrum of ES, identifying several genes that are involved in the malignant pathways specific to the growth of ES cells [9–15]. Gene expression profiles after the modulation of EWS/FLI1 levels have shown TGFBR2, IGFBP3, DKK1, NKX2.2, NR0B1 and some genes as EWS/FLI1 targets [16, 17]. These comprehensive studies have improved our understanding of the biology of ES and may lead to the development of practical tumor markers to support individualized therapy. In addition, a proteomic study reported that the expression of NPM1 is correlated with a poor prognosis in ES [18]. However, the protein expression profiles associated with EWS/FLI1 have yet to be elucidated.

In this study, to identify the regulated proteins associated with EWS/FLI1, elucidate the function of EWS/FLI1 and identify the therapeutic targets, we conducted proteomic studies using an EWS/FLI1 knockdown in ES cell lines and human mesenchymal stem cells (hMSCs) expressing EWS/FLI1. We also conducted network analyses based on the protein profiles and carried out functional analyses of an identified critical pathway both *in vitro* and *in vivo* with inhibitors.

## RESULTS

### EWS/FLI1 knockdown in ES cell lines

**Figure 1:**
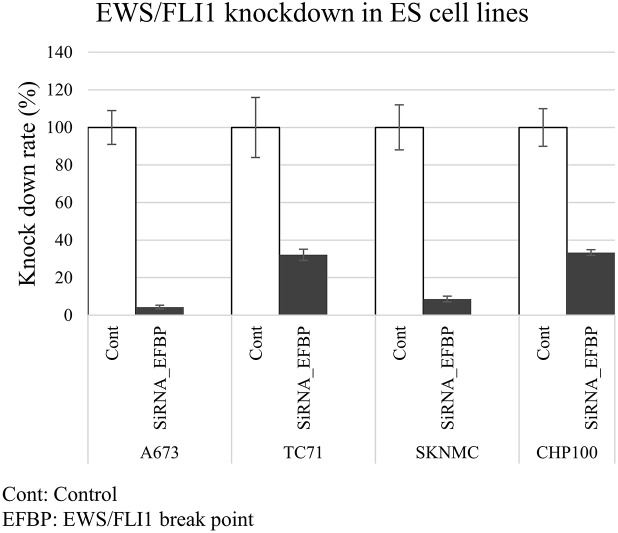
Expression of EWS/FLI1 in ES cell lines Proteomic studies were performed using proteins extracted from four ES cell lines (type 1: A673, TC71and SKNMC, type 2: CHP100) that were transfected with siRNAs targeting EWS/FLI1 break point (EFBP). The quantitative PCR (qPCR) assays showed that EFBP siRNA inhibited the mRNA expression of EWS/FLI1 in all four ES cell lines.

EWS/FLI1 knockdown was performed using two siRNA designed for the EWS/FLI1 break point (EFBP type 1 for A673, TC71 and SKNMC; EFBP type 2 for CHP100) (Supplementary Figure 1). Both EFBP siRNAs inhibited EWS/FLI1 protein and mRNA expression in A673, TC71, SKNMC and CHP100 (Figure 1 and Supplementary Figure 1A). With regard to cell proliferation, EWS/FLI1 knockdown with both siRNAs efficiently suppressed the cell proliferation of A673, TC71, SKNMC and CHP100 (Supplementary Figure 1B).

### EWS/FLI1 overexpression in hMSCs

The expression of EWS/FLI1 in the three hMSC cell lines was induced using the Retroviral Gene Transfer and Expression kit (Clontech Laboratories, Inc., CA, USA), in accordance with the manufacturer's recommendations. We confirmed the expression of the EWS/FLI1 fusion genes in hMSCs using reverse transcription polymerase chain reaction (RT-PCR) (Supplementary Figure 2).

### Identification of protein profiles associated with EWS/FLI1 expression in both ES cell lines and hMSCs by i-TRAQ

To identify protein profiles associated with EWS/FLI1, we performed i-TRAQ analyses using ES cells transfected with siRNA EFBP1 type 1, type 2 or control (A673, TC71, SKNMC and CHP100) and hMSCs transfected with EWS/FLI1 vector and retroviral gene transfers. Proteins were extracted from both transfected ES cells and hMSCs. We performed isobaric tags for relative and absolute quantitation (i-TRAQ) analyses using each cell and identified 1500-2200 proteins in each analysis. Statistical comparison led to the compilation of the protein profile, which differed significantly between the siRNA targets (EFBP1 types 1 and 2) and control as well as between hMSCs expressing EWS/FLI1 and control hMSCs.

**Figure 2:**
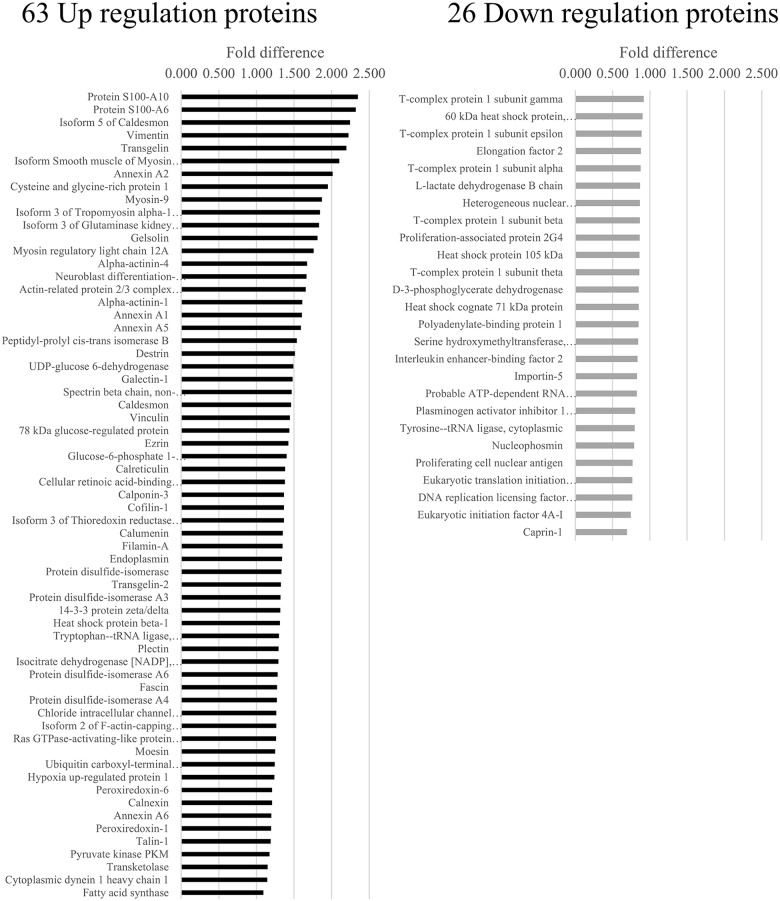
Protein profiles of EWS/FLI1 knockdown in 4 Ewing sarcoma cell lines Isobaric tags for relative and absolute quantitation (i-TRAQ) analyses identified more than 2,000 proteins regulated by the EWS/FLI1 fusion as well as 63 consistently upregulated and 26 consistently downregulated proteins that were commonly altered in all four ES cell lines.

In ES cells with knockdown of EWS/FLl1 (Figure 2 and Supplementary Tables 1–4), analyses showed 31 downregulated proteins associated with EWS/FLI1 knockdown in A673, 37 in TC71, 57 in SKNMC and 17 in CHP100 (p < 0.05). Furthermore, analyses showed 74 upregulated proteins associated with EWS/FLI1 knockdown in A673, 70 in TC71, 75 in SKNMC and 43 in CHP100 (p < 0.05). We analyzed the four profiles to identify proteins that were similarly altered in all four cell lines and found 63 consistently upregulated and 26 consistently downregulated proteins.

In hMSCs expressing EWS/FLI1 (Supplementary Tables 5–8), analyses showed 62 downregulated proteins associated with EWS/FLI1 overexpression in hMSC1, 60 in hMSC2 and 67 in hMSC3 (p < 0.05). Furthermore, analyses showed 72 upregulated proteins associated with EWS/FLI1 overexpression hMSC1, 43 in hMSC2 and 59 in hMSC3 (p < 0.05). We analyzed the four profiles to identify proteins that were similarly altered in all three cell lines and found 31 consistently upregulated and 19 consistently downregulated proteins.

### Network analysis according to the protein profiles of EWS/FLI1

**Figure 3:**
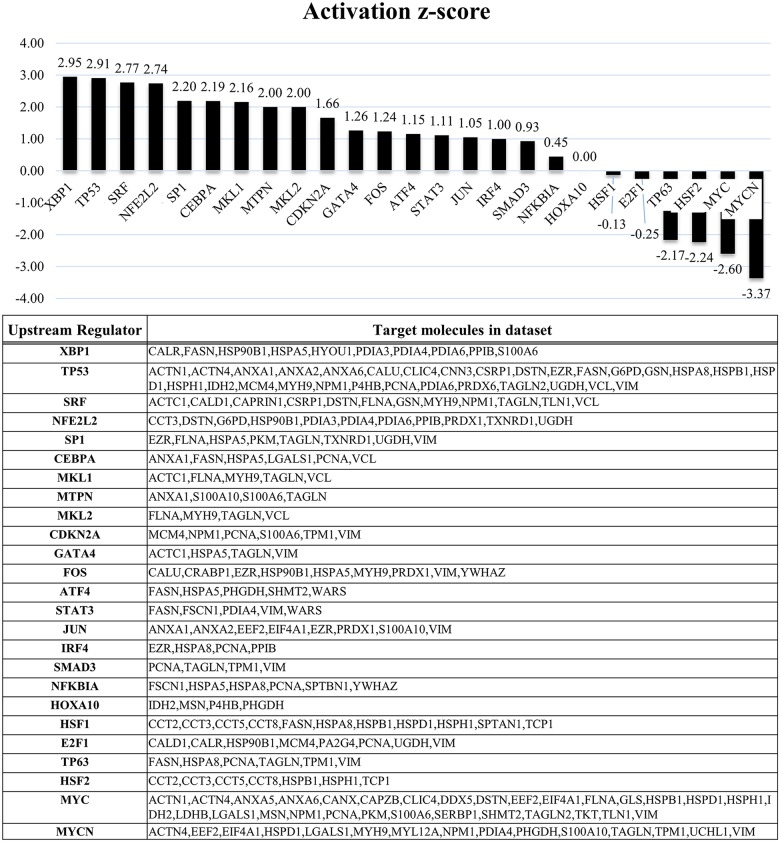
IPA analyses based on the protein profile of EWS/FLI1 In addition, the network analyses identified several critical pathways, including XBP1, which was ranked the highest. XBP1 is a protein well known to play an important role in the unfolded protein response (UPR) to endoplasmic reticulum (ER) stress through the IRE1α-XBP1 pathway. And TP53 also was ranked at second highest.

To further understand the biological processes and networks involved in tumor malignancy based on the proteins regulated by EWS/FLI1, we performed network analyses using the Ingenuity Pathways Analysis (IPA) system (IPA; QIAGEN, Redwood City, CA, USA). We conducted analyses using i) the proteins associated with EWS/FLI1 knockdown in ES cell lines and ii) the proteins associated with hMSCs expressing EWS/FLI1. In these independent network analyses based on the profiles of EWS/FLI1 knockdown in ES cells, we identified several pathways involving XBP1 that played a critical functional role and acted as upstream regulators of these proteins known to be associated with EWS/FLI1 expressions in ES cells (Figure 3). Based on a network analysis indicating that XBP1 was ranked highest among these identified pathways, we conducted a functional analysis of the XBP1 pathway in order to identify any possible therapeutic targets.

### Expression of XBP1u (unspliced XBP1) and XBP1s (spliced XBP1) in ES cell lines and surgical materials

**Figure 4:**
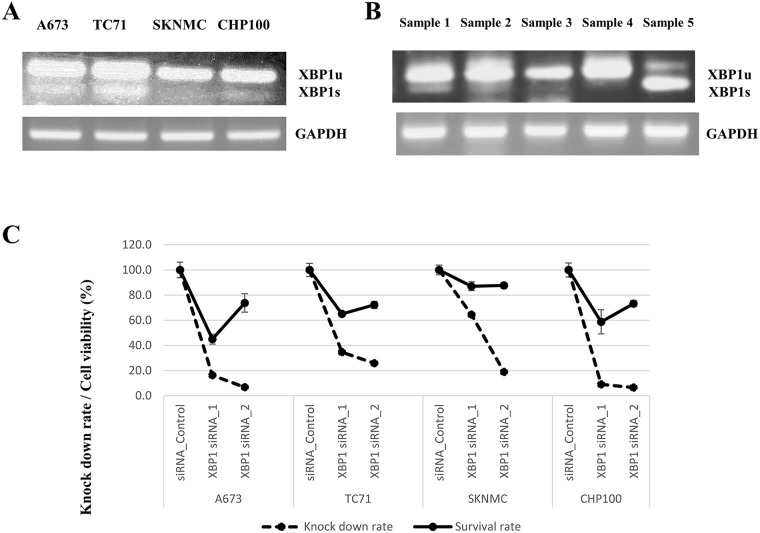
**(A and B)** Expression of XBP1u (XBP1 unspliced) and XBP1s (XBP1 spliced). **(C)** Cell viability following XBP1 knockdown in ES cell lines. (A, B) The mRNA expression of both XBP1u and XBP1s in four ES cell lines (type 1: A673, TC71 and SKNMC, type 2: CHP100) and five ES surgical materials was measured by RT-PCR. The activated IRE1α endoribonuclease removes a 26-nucleotide intron from XBP1, and the translational frame-shift changes unspliced XBP1 (XBP1u: inactive) into spliced XBP1 (XBP1s: active) [30]. (A) Among the ES cell lines, TC71 and A673 had a high mRNA expression of XBP1s, while SKNMC and CHP100 had a low expression of XBP1s. (B) Among the surgical materials of ES, sample 1 had EWS/FLI- type 2 fusion gene, and samples 2-5 had EWS/FLI- type 1 fusion gene. Samples 1, 2 and 5 had high expression of XBP1s (type 1: 2/4 [50%] and type 2 1/1 [100%]). (C) XBP1 siRNA knockdown in ES cell lines (A673, TC71, SKNMC and CHP100) was performed to verify the associations between the XBP1 expression and cell viability. XBP1 siRNA suppressed the expression of XBP1 in ES cell lines, as shown by q-PCR. Regarding cell proliferation, silencing XBP1 inhibited the cell viabilities in all three ES cell lines.

To confirm the expression of XBP1 in ESs, the expression of XBP1u and XBP1s in four ES cell lines and five surgical materials was verified. In ES cell lines, PT-PCR (reverse transcription-polymerase chain reaction) of XBP1s demonstrated that A673 (type 1) and TC71 (type 1) had high expression, while SKNMC (type 1) and CHP100 (type 2) had low expression according to the mRNA levels (Figure 4A). Regarding the surgical materials, 3 of 5 samples (60%: sample 1, 2 and 5) showed a high expression of XBP1s (Figure 4B and Supplementary Table 9). With respect to the subtype of EWS/FLI1 fusion, 2 of 4 samples (50%) for type 1 and 1 of 1 sample (100%) for type 2 had XBP1s expressions (Figure 4B and Supplementary Table 9).

### Effects of silencing XBP1 on the viability of ES cells

To investigate the association between the IRE1α-XBP1 pathway and the viabilities of ES cells, inhibition of XBP1 was performed via XBP1 siRNA knockdown in 4 ES cell lines (A673, TC71, SKNMC and CHP100). q-PCR (quantitative polymerase chain reaction) revealed significant inhibitions of total XBP1 mRNA in ES cells, including A673, TC71, SKNMC and CHP100 (Figure 4C). In the cell proliferation assays, we also confirmed that the cell viabilities were suppressed in ES cells including A673, TC71, SKNMC and CHP100 by silencing XBP1 expression (Figure 4C). These results revealed that the inhibition of XBP1 significantly suppressed cell growth and associations between the XBP1 expression and tumor malignancies in ES cells.

### Activity of IRE1α-XBP1 inhibitors in ES cells

**Figure 5:**
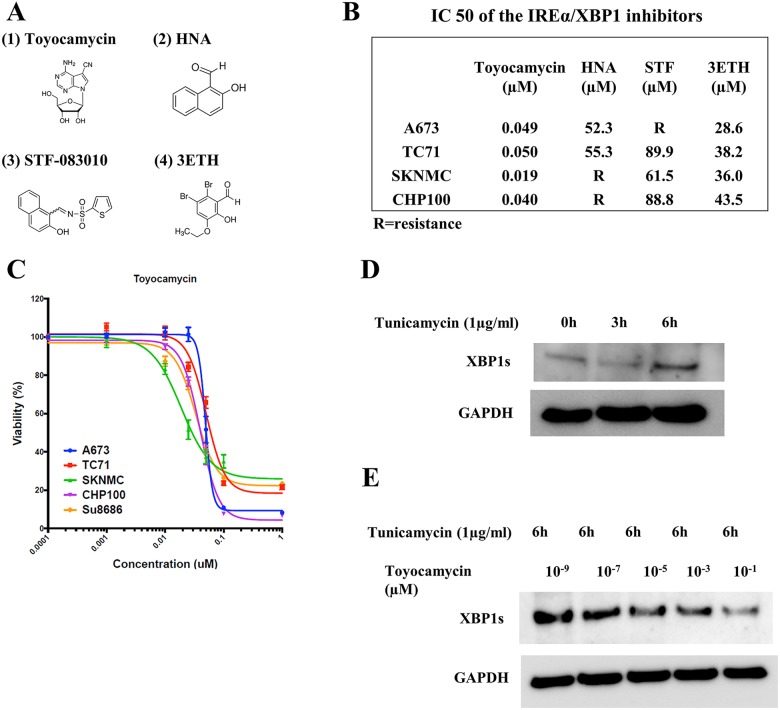
The activity of IRE1α-XBP1 pathway inhibitors in ES cells **(A)** The chemical formulas of four types of IRE1α-XBP1 pathway inhibitors. (1) Toyocamycin, (2) 2-hydroxy-1-naphthaldehyde (HNA), (3) STF-083010 (STF) and (4) 3-ethoxy-5 6-dilbromosalicylaldehyde (3ETH). These chemical formulas are totally different from one another. **(B)** The IC50 values of IRE1α-XBP1 inhibitors. The IC50 values of the four inhibitors were measured in four ES cell lines. Toyocamycin had the highest inhibition of ES cell growths among the inhibitors. **(C)** Cell viability curve of toyocamycin in ES and Su8686 (positive control cell line). The cell viabilities of toyocamycin were investigated in four ES cell lines and Su8686 (pancreatic cancer) which have high sensitivities of toyocamycin according to published articles. Toyocamycin significantly and dose-dependently inhibited the cell viabilities in all ES cell lines. **(D)** Tunicamycin (TM) mediated the expression of XBP1s in an ES cell line (TC71). Western blotting showed that TM stimulation time-dependently enhanced the expression of XBP1s in the TC71 cell line. **(E)** Toyocamycin inhibited XBP1s expression. Under conditions of TM stimulation, toyocamycin inhibited XBP1 expression dose-dependently.

Recently, a number of IRE1α-XBP1 inhibitors, such as toyocamycin, 2-hydroxy-1-naphthaldehyde (HNA), STF-083010 (STF) and 3-ethoxy-5 6-dilbromosalicylaldehyde (3ETH), have been developed [19] (Figure 5A). We therefore investigated the inhibition abilities of these four IRE1α-XBP1 inhibitors in ES cells. Toyocamycin inhibited the cellular viability of all ES cell lines with the IC50 values shown in Figure 5B. In HNA, STF and 3ETH, these inhibitors either partly suppressed or didn't suppress the cell viabilities in ES cell lines (Figure 5A and 5B). The treatment of ES cells with increasing dosages showed that toyocamycin exerted the most effective tumor suppression among the four regents (Figure 5A–5C). Regarding IC50 of toyocamycin, all ES cell lines demonstrated <0.050 μM (low concentrations). Furthermore, at 1μM (final concentrations), CHP100 (6.7%), A673 (8.3%), TC71 (21.5%) and SKNMC (22.3%) had lower cell viabilities than in Su8686 (control cell: pancreatic adenocarcinoma cell line and high sensitive to toyocamycin) (23.3%) (Figure 5C). Based on the findings of other studies investigating the effect of IRE1α-XBP1 inhibitors on other cancer types and our own results, we employed toyocamycin in subsequent assays.

### Effects of toyocamycin on the expression of XBP1s and viability of ES cells

Tunicamycin (TM) generally induces ER stress and XBP1s production [19, 20]. TM inhibits protein glycosylation in the ER and leads to ER stress [19, 20]. Increased expression of XBP1s and decreased expression of XBP1u have been identified following TM treatment [19, 20]. In our assays using ES cells, TM stimulation (1 μg/ml, 6 h) increased the expressions of XBP1s in TC71 cells (Figure 5D). We therefore examined the inhibitory abilities of toyocamycin on XBP1s induced by TM. Toyocamycin inhibited the expression of XBP1s induced by TM in a dose-dependent manner (range: 10^−9^ to 10^−1^ μM) (Figure 5E). These results indicate that toyocamycin exerts anti-tumor activity through the IRE1α-XBP1 pathway in ES cells.

### Effects of toyocamycin on the viability of ES cells by activating apoptosis

**Figure 6:**
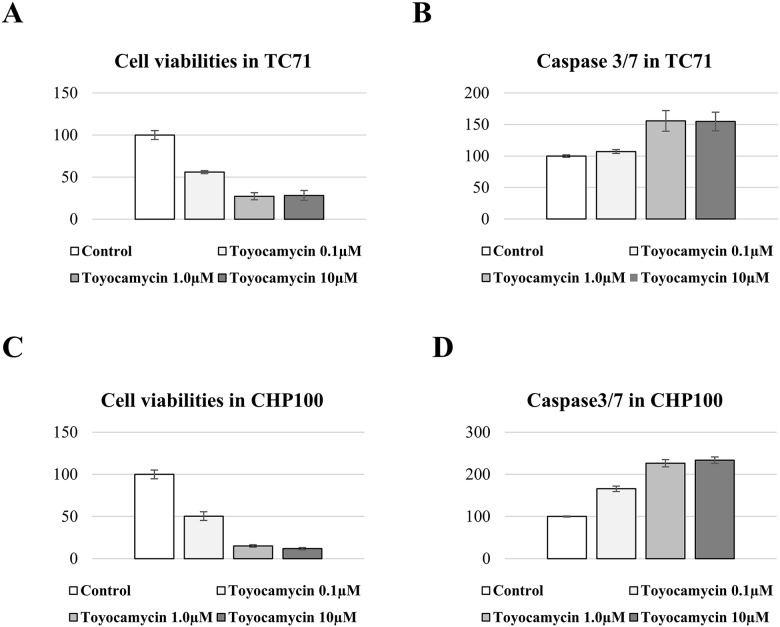
Toyocamycin activated apoptosis and reduced the cell viability of ES cell lines The cell viability of TC71 was significantly and dose-dependently suppressed by toyocamycin **(A)**. Toyocamycin activated Caspase3/7 activity in TC71 with decreasing cell viability **(B)**. The cell viability of CHP100 was significantly and dose-dependently inhibited by toyocamycin **(C)**. Toyocamycin enhanced the Caspase3/7 activity in CHP100 with decreasing cell viability **(D)**.

In Caspase-3/7 assays, cell proliferation and the activation of Caspase-3/7 were measured in ES cells (TC71 and CHP100) treated with toyocamycin. These assays revealed that toyocamycin inhibited the tumor cell viabilities and activated the expression of Caspase-3/7 in all ES cells (Figure 6A–6D). These results indicated that toyocamycin inhibited cell viabilities by activating apoptosis in ES cells.

### Anti-tumor activities of toyocamycin on ES cells *in vivo*

**Figure 7:**
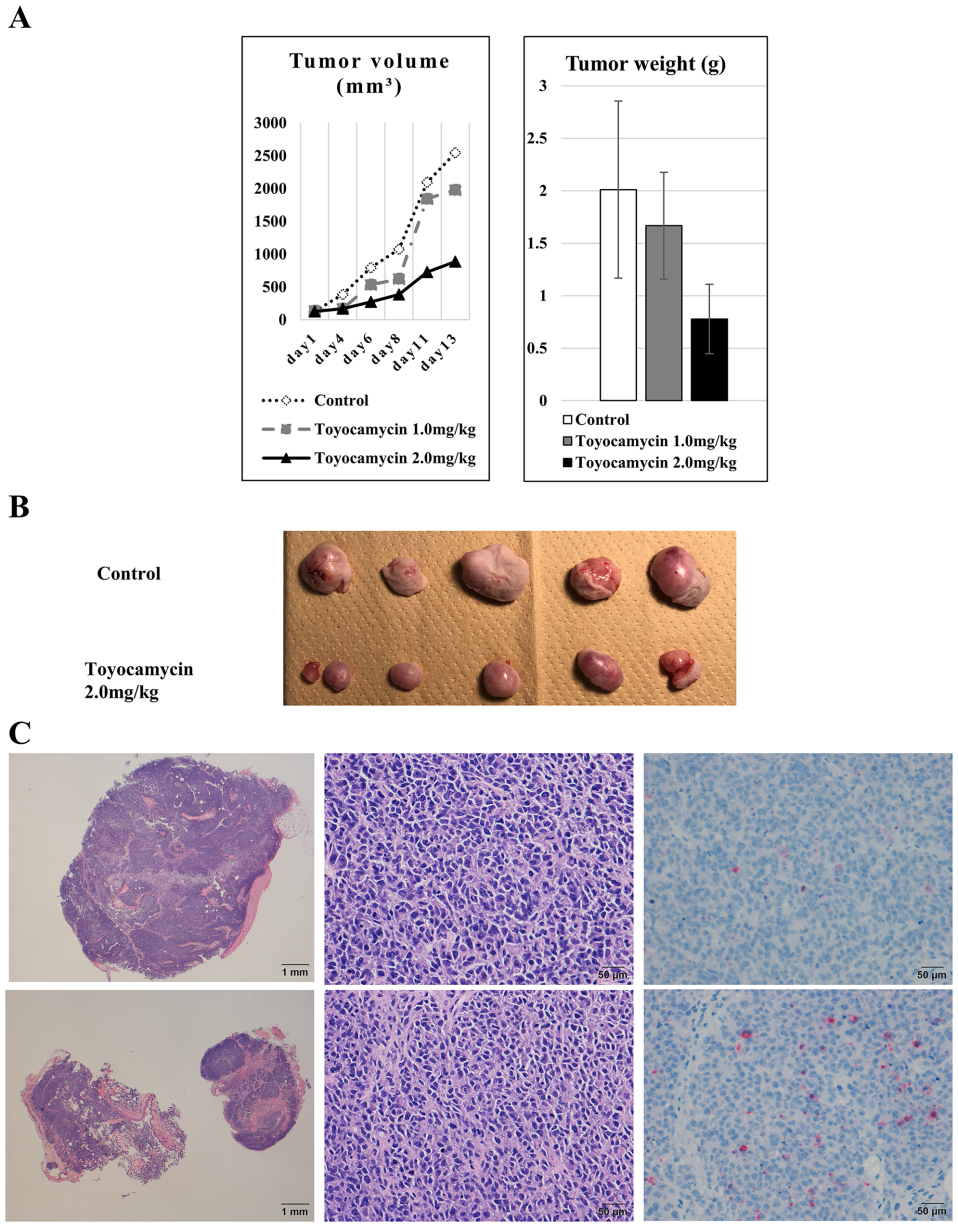
Toyocamycin exerted significant anti-tumor activity against ES cells in *in vivo* assays To verify the anti-tumor activity of IRE1α-XBP1 inhibitors, *in vivo* assays were performed using mice and ES cells. **(A)** Regarding the associations between the inhibitors and tumor size (including volume and weight) in mice, toyocamycin dose-dependently inhibited both the tumor volume and weight compared to control animals (tumor volume: p = 0.078 and tumor weight: p = 0.025). **(B)** Gross features of tumors. Tumors treated with inhibitors were smaller than control ones. **(C)** We confirmed the tumor mass in mice by HE staining. These results demonstrated that toyocamycin exerts anti-tumor activity of ES cells *in vivo*. Microscopic features on HE (left and middle panels) and cleaved caspase-3 staining (right panels) of tumors treated with toyocamycin (lower panels) and control (upper panels). An examination of the HE-stained features at low power under a microscope (left panels) showed that the tumors treated with toyocamycin (left upper panel) were smaller than the control ones (left lower panel). An examination of the HE-stained features at high power indicated that the masses consisted of tumor cells in both toyocamycin-treated (middle lower panel) and control tumors (middle upper panel). The microscopic features on cleaved caspase-3 staining (right panels) indicated that the tumors treated with toyocamycin (right lower panel) had higher numbers of stained cells than control tumors (right upper panel).

To evaluate the anti-tumor activities of toyocamycin in ES cells *in vivo*, BALB/c nude mice (8 weeks of age, female) inoculated subcutaneously with ES cells (A673) were administered toyocamycin intraperitoneally once-weekly at 1.0 or 2.0 mg/kg. We measured the tumor volumes three times a week with digital calipers and calculated tumor volumes using the modified ellipsoid formula 1/2 (Length × width^2^) [21, 22]. In *in vivo* assays, mice treated with toyocamycin had significantly smaller tumor volumes (887 mm^3^ vs. 2546 mm^3^, p=0.078) and lighter tumor weights (0.778 g vs. 2.01 g, p=0.025) than control mice at day 13 (Figures 7A and 7B). These tumors in mice were verified by HE (hematoxylin and eosin) and cleaved caspase-3 staining to confirm the tumor size, cell viabilities and apoptosis activities. Regarding the tumor size, the tumors that received the toyocamycin treatment were smaller than those in controls on HE staining (Figure 7C). We also confirmed that the masses consisted of tumor cells in both toyocamycin treated and control animals (Figure 7C). On cleaved caspase-3 staining, the tumors treated with toyocamycin had higher numbers of stained cells than controls (Figure 7C). These results indicated that toyocamycin exerted its anti-tumor activity by enhancing cell apoptosis.

## DISCUSSION

We conducted a proteomic study focusing on EWS/FLI1 fusion in ES and successfully identified several characteristic proteins as being associated with EWS/FLI1 expression. To our knowledge, this study is the first regarding the protein expression of EWS/FLI1 fusion in ES.

Our protein profiles consistently identified 90 proteins regulated by EWS/FLI1 expression across four ES cell lines. A proteomic study using ES surgical samples identified high protein expression of NPM1 and confirmed that the expression was significantly associated with tumor malignancy and the prognosis in ES patients [18, 23]. In our profiles of proteins associated with the inhibition of EWS/FLI1, NPM1 was also identified as a downregulated protein. To verify the accuracy of our protein profiles, we conducted western blotting using proteins transfected with EWS/FLI1 siRNA knockdown in ES cell lines. In the assay, the low expression of NPM1 was observed in EWS/FLI1 knockdown proteins (Supplementary Figure 1). These results indicated that our established protein profiles depicted the protein expression accurately. Furthermore, to confirm whether our protein profiles were significantly associated with EWS/FLI1 onco-protein and tumor malignancy, we performed cell proliferation assays based on either NPM1 or EWS/FLI1 siRNA knockdown. We found that silencing EWS/FLI1 reduced the viability of ES cell lines (Supplementary Figure 1), as did inhibiting NPM1 (data not shown). These findings suggest that our protein profiles for EWS/FLI1 consisted of accurate information.

In network analysis according to the protein profiles of EWS/FLI1, XBP1 was ranked at highest among these identified pathways. And TP53 also was ranked at second highest (Figure 3). With respect to TP53, several articles described that p53 gene alterations have been detected in approximately 10% of ESs and these alterations were also associated with highly aggressive behavior and poor chemo response in ESs [4, 24, 25]. These findings also suggest that our network analyses and protein profiles consisted of accurate information.

Recent studies have suggested that XBP1 is a basic region/leucine zipper (bZIP) transcription factor of the CREB-ATF family. In addition, IRE1α-XBP1 is one of the main pathways of the UPR to ER stress [20]. The endoplasmic reticulum (ER) is the main subcellular compartment involved in protein folding and the maintenance of cellular homeostasis [26, 27]. In mammalian cells, protein folding plays crucial roles in maintaining proportionally fine-tuned cells and it also allows the cells to adapt to the microenvironment via the metabolic state in the cells [20]. Extracellular stimulation, including low nutrient levels (narrow space), hypoxia (narrow space) and exposure to multiple drugs (therapy), induces the accumulation of misfolded proteins in the ER, thereby causing ER stress and initiating the UPR [5]. The UPR activates the biosynthetic capacity of ER stress and decrease the biosynthetic burden of ER stress in order to maintain cellular homeostasis. Under conditions of such prolonged and uncompensated ER stress, the potential UPR leads to difficulty in handling ER stress. As a result, cellular apoptosis finally occurs [28].

The UPR consists of three main signaling pathways: inositol-requiring enzyme 1α (IRE1α), PKR-like ER kinase (PERK) and activating transcription factor 6 (ATF6) [29]. Regarding IRE1α-XBP1 in the UPR, IRE1α initiates the splicing of XBP1 mRNA as IRE1 endoribonucleases is activated by oligomerization and autophosphorylation. The activated IRE1α endoribonuclease removes a 26-nucleotide intron from XBP1, and the translational frame-shift changes unspliced XBP1 (XBP1u: inactive) into spliced XBP1 (XBP1s: active) [30]. Several studies have revealed an association between the IRE1α-XBP1 pathway and human cancers [31–33]. This pathway plays a critical role as a part of the ER stress response in tumor growth, clinical outcome and chemo-resistance [20, 29, 34, 35]. In particular, in multiple myeloma (MM), several reports have revealed the overexpression of XBP1 and its involvement in the pathogenesis [36, 37]. Regarding the involvement of XBP1, it was suspected that MM cells are under chronic ER stress conditions in an effort to survive; this chronic stress initiates the UPR, including activation of the IRE1a-XBP1 pathway. Furthermore, MM cells are located in the bone marrow, which is usually considered an environment of few nutrients (narrow space) and hypoxia (narrow space) compared to other organs [38, 39]. Of note: ES also mainly occurs in the bone marrow and so flourishes under similar conditions to MM.

Recent studies have focused on the pathogenesis of ER stress after adaption by the UPR in several cancers, because this mechanism may be useful for developing new therapeutic strategies for targeting signaling of the UPR and inhibiting this key survival pathway, including the IRE1α-XBP1 pathway. Functional studies have conducted inhibitor assays in several malignant tumors, including MM [20, 40], pancreatic cancer [19], ovarian cancer [41], and prostate cancer [42] using IRE1α-XBP1 inhibitors both *in vitro* and *in vivo*. Their findings indicated that these inhibitors successfully exerted anti-tumor activities [20, 27]. In particular, IRE1α-XBP1 has been used in combination therapies with conventional chemotherapy because the signaling of the UPR has completely different pathways in comparison to conventional drugs. Furthermore, chemotherapies usually enhance the ER stress and the UPR has thus been elucidated by functional analyses using cell lines [20].

Given that the overexpression of XBP1s indicates the activation of the UPR and excess ER stress in cells, we verified the expression of XBP1s in four ES cell lines and five surgical materials. For EWS/FLI1 type 1 fusion, 2 of 3 (66%) ES cell lines and 2 of 4 (50%) surgical samples showed overexpression of XBP1s (high expression: A673 and TC71, weak expression: SKNMC). In contrast, for EWS/FLI1 type 2 fusion, we observed weak XBP1s expression in one cell line (CHP100; 0%) and high expression in one surgical sample (100%). Regarding the protein levels, we confirmed the time-dependent increased expression of XBP1s with TM stimulation in ES cell lines. Therefore, based on our results, almost half of all ES cases demonstrated the expression of XBP1s. Furthermore, although we examined the XBP1s expression in only a few cases in our series, further studies using larger cohorts might be able to clarify these associations between the clinicopathological factors, including fusion types, and the expression of XBP1.

Regarding the association of XBP1s expression with cell proliferation, we assessed the relationship between XBP1s and cell viability in EWS/FLI1 type 1 fusion (A673, TC71 and SKNMC). Silencing XBP1 led to a more significant inhibition of cell growth in the higher XBP1s expression group (A673 and TC71) than in the lower XBP1s expression group (SKNMC). The cell viabilities showed that A673 was 45.1%, TC71was 65.1% and SKNMC was 87.1%. In the type 2 fusion of EWS/FLI1 (CHP100), the inhibition of the XBP1 expression also suppressed the viability of CHP100 cells; the XBP1s expression of these cells was low. In the type 1 fusion, these results indicated that the UPR, including the expression of XBP1, played a significant role in the tumor activities of ES cells. In inhibitor assays using toyocamycin in type 1 fusion, the cell viabilities of the high XBP1s expression group (A673 and TC71) demonstrated superior cell inhibition compared with SKNMC (low XBP1s expression) at a final concentration of 1 μM (Figure 5C). We also confirmed that toyocamycin dose-dependently inhibited the XBP1s expression induced by TM and activated apoptosis in TC71 (Figure 5D and 5E). These results indicated that toyocamycin suppressed tumor growth by inhibiting XBP1s and the IRE1α-XBP1 pathway in ES cells. However, the SKNMC cell line was also sensitive to toyocamycin in comparison to su8686 (control cells) (Figure 5C). Furthermore, in CHP100 (the type 2 fusion of EWS/FLI1) which had a low XBP1 expression, toyocamycin exerted significant anti-tumor activities and also activated apoptosis. Based on our results, in ES, IRE1α-XBP1 inhibitors, especially toyocamycin, acceptably exert anti-tumor activity regardless of whether these tumors had a high or weak expression of XBP1s.

Several studies have explored the efficacy of targeting ER stress and the UPR in several tumors using IRE1α-XBP1 inhibitors. Functional assays conducted in MM, CML (chronic myeloid leukemia), pancreatic cancer and prostate cancer both *in vitro* and *in vivo* showed that toyocamycin exerted significant anti-tumor activities in these tumors [19, 20, 26, 42–44], findings which we verified in the present studies in ES cells. In addition, several articles have similarly reported that other IRE1α-XBP1 inhibitors, including STF, HNA and 3ETH, significantly inhibited several types of tumors [19, 27, 40]. We also verified the anti-tumor activities of other IRE1α-XBP1 inhibitors, including STF, HNA and 3ETH, in ES cell lines. However, these inhibitors did not have any significant anti-tumor activities in ES cell lines. Further studies might clarify the mechanism underlying these XBP1 inhibitors’ activities in ES cell lines.

With respect to the anti-tumor functions of toyocamycin, some reports noted that toyocamycin inhibited XBP1 splicing via a conformational change in IRE1α to attenuate the activation of XBP1 [20, 45]. One study reported that toyocamycin did not cause IRE1α phosphorylation, but specifically blocked its endoribonuclease activity [27]. Furthermore, toyocamycin did not affect ATF6α or PERK signaling [27]. Based on these conflicting reports, the mechanisms underlying the effects of toyocamycin remain unknown. We think that these anti-tumor mechanisms of toyocamycin will be elucidated in further studies.

With respect to the toxicities of toyocamycin, an earlier phase I toyocamycin single-agent study (NSC-63701) testing the possible anti-tumor effects in patients with advanced solid tumors was planned in 1968; there has been no further clinical evaluation [46]. Recently, druggable effects have been reported in several types of cancer and chronic disease [20, 27, 45]. However, these were basic studies, and no clinical trials have been reported. In the future, the anti-tumor activity of toyocamycin and adverse events associated with its administration may be revealed from clinical data.

Furthermore, chemoresistance associated with the expression of XBP1s has been identified in MM, so some studies have administered combination therapies including IRE1α-XBP1 inhibitors [20]. Based on a previous article study on combination therapies, we conducted combination assays to investigate the combined effects of doxorubicin and toyocamycin in ES cell lines. The results showed that the combination of these drugs inhibited cell viability in a dose-dependent manner (Supplementary Figure 3). The present and previous findings therefore show that IRE1α-XBP1 inhibitors exert acceptable anti-tumor activities against malignant tumors.

We conducted proteomic analyses of EWS/FLI1 associated with ES. Our data suggest that the growth of ES cells may be enhanced by the physiological association between the identified proteins and EWS/FLI1. We believe that our established protein profiles will help improve our understanding of the relationship between EWS/FLI1 and malignant behavior in patients with ES and may lead to the development of novel therapeutic strategies. In this study, we also found that IRE1α-XBP1 inhibitors exerted anti-tumor activity against ES, in part through their inhibition of the UPR and IRE1α-XBP1 pathway. These findings suggest that XBP1 is likely a novel target in ES cells. Future studies should explore the function of XBP1 in ES in greater detail and examine the associations between EWS/FLI1 and XBP1, along with their roles in ER stress and the UPR in ES. These studies should also confirm through additional further functional assays using combination therapy consisting of toyocamycin and conventional drugs for ES treatment whether or not such combination therapies enhance the anti-tumor activities against ES. Taken together, our present findings suggest that IRE1α-XBP1 inhibitors may be useful therapeutic options for ES patients and might improve the existing therapeutic strategies and outcomes for patients with this disease.

## MATERIALS AND METHODS

### Cell lines and surgical materials

A673 and SK-N-MC were obtained from American Type Culture Collection (ATCC). CHP-100 and TC-71 were provided by Dr. Melinda Merchant (National Cancer Institute, Bethesda, MD, USA). The TC71, SKNMC and CHP100 cell lines were grown in Roswell Park Memorial Institute (RPMI) medium supplemented with 10% FCS and penicillin/streptomycin. The A673 cell line was grown in Dulbecco's modified Eagle's medium (DMEM) supplemented with 15% FCS and penicillin/streptomycin. Human mesenchymal stem cells were purchased from Lonza (#PT-2501, Walkersville, MD, USA) and from ATCC (#PCS-500-012; Manassas, VA, USA). ATCC #PCS-500-012 Lot#61808864 is from a 20-year-old African American male and ATCC #PCS-500-012 Lot#0202 is from a 32-year-old Latino or Hispanic female. hMSCs were cultured in mesenchymal stem cell media (Lonza, Walkersville, MD, USA, #PT-3001), and cell propagation was limited to 7 passages before immortalization. Five samples of ES surgical materials that consisted of EWS/FLI1 type 1 (four samples) and type 2 (one sample) were employed.

### RNA extraction and RT-PCR (reverse transcription-polymerase chain reaction)

RNA was extracted using RNeasy Plus Mini kit (Qiagen. Hilden, Germany), and first strand synthesis was performed using 5 μg of RNA and the SuperScript^®^ IV First-Strand Synthesis System (Thermo Fisher Scientific, Commonwealth, MA, USA). We performed RT-PCR analyses to evaluate the expression of EWS/FLI1 and XBP1u/XBP1s using PCR SuperMix (Thermo Fisher Scientific). The human EWS/FLI1 primer sequences were as follows: 5’- CCAAGTCAATATAGCCAACAG −3’ and 5’- GGCCAGAATTCATGTTATTGC −3’. The human XBP1 primer sequences were as follows: 5’-CCTGGTTGCTGAAGAGGAGG-3’ and 5’-CCAT GGGGAGATGTTCTGGAG-3’. GAPDH was used as a loading control, with primers as follows: 5’-GAAG GTGAAGGTCGGAGTC3’ and 5’- GAAGATGGT GATGGGATTT-3’.

### Western blotting analyses

The protein samples were separated by SDS-PAGE and subsequently blotted on a nitrocellulose membrane. The membrane was incubated with antibodies against Fli-1 (C-19 rabbit polyclonal, 1:200 dilution, Santa Cruz, CA, USA), XBP-1s (D2C1F Rabbit mAb, 1:1000 dilution; Cell Signaling, MA, USA) or GAPDH (GTX627408, 1:1000 dilution; GeneTex, CA, USA ), and then with horseradish peroxidase-conjugated secondary mouse antibody (1:1000 dilution; GE Healthcare Biosciences. Little Chalfont, UK) or rabbit antibody (1:1000 dilution; GE Healthcare Biosciences). The expression of the proteins was detected using Super Signal West Pico Chemiluminescent Substrate (Thermo Fisher Scientific) autoradiograms.

### Quantitative real-time PCR

Quantitative real-time PCR (qPCR) was performed using inventoried Taqman assays from Applied Biosystems, CA, USA) (20x Primer Probe mix) corresponding to EWSR1 (Assay ID Hs03024497_ft), ACTB (Assay ID Hs01060665_g1) and GAPDH (Assay ID Hs02758991_g1). XBP1. All PCR reactions were performed with TaqMan Fast Advanced Master Mix (Applied Biosystems) on an Applied Biosystems Step One Plus Real Time PCR System in accordance with the standard protocols. The amount of each target gene relative to the housekeeping gene 18S, ACTB and GAPDH was determined using the comparative threshold cycle (Ct) method. For each sample, the relative amount of each target gene was calibrated against a control sh-RNA transfected cell line (Cont). All assays were performed in triplicate.

### EWS/FLI1 siRNA and XBP1 siRNA knockdown in ES cell lines

For the knockdown expression studies, we used four cell lines (type 1: A673, TC71, SKNMC and type 2: CHP100). ES cell lines expressing wild-type EWS/FLI1 were treated with 50 nM of either EWS/FLI1 break-point siRNA for type 1 (Sense: 5′-GCAGCAGAACCCUUCUUAUUU-3’, Antisense: 3′-UUCGUCGUCUUGGGAAGAAUA-5′), EWS/FLI1 break-point siRNA for type 2 (Sense: 5′-GGCAGCAGAGUUCACUGCUUU-3′, Antisense: 3′-UUCCGUCGUCUCAAGUGACGA-5′) or siRNA negative control (Sigma-Aldrich. MO, USA) using Lipofectamine™ RNAiMAX reagent (Thermo Fisher Scientific). XBP1 siRNA knockdown was also performed using pre-designed XBP1 siRNA (sc-38627, Santa Cruz; or siRNA negative control, Sigma-Aldrich). After 72 h, the total protein and RNA from each cell line was isolated, and the expression was validated by western blotting analyses and reverse transcriptase PCR (RT-PCR).

### Preparation of retrovirus and transduction of cell lines

For retrovirus production, the pcx4 (PMID: 14597713) and pBabe vectors system were used. pBabe-hygro-hTERT (Addgene plasmid 1773) was obtained from Addgene (Cambridge, MA, USA). pcx4-bsr-SV40ER was provided by Drs. Tsuyoshi Akagi and Ken Sasai (both at KAN Research Institute, Inc., Kobe, Japan). EWS/FLI1 was subcloned into pcx4-bleo. Retroviruses were obtained using 293T cells as packaging cells and infected into hMSC lines and selected with 200 μg/ml hygromycin B, 10 μg/ml blasticidin, or 500 μg/ml zeocin (Thermo Fisher Scientific).

### i-TRAQ sample labeling, mass spectrometry analyses and peptide identification

Analyses of proteins by isobaric tags for relative and absolute quantification (i-TRAQ), a chemical label detected by mass spectrometry, were performed as described in our previous article [47]. Briefly, cell lysate samples were concentrated, and was buffer exchanged using 3.5-kDa molecular weight cut-off spin concentrators (Tomy Seiko Co., Ltd., Tokyo, Japan) and then digested overnight with 10 μg L-1-(4-tosylamido)-2-phenylethyl tosylphenylalanyl chloromethyl ketone-treated trypsin. Each peptide solution was labeled with 1 of the 8 iTRAQ reagents (iTRAQ reporter ions of 113, 114, 115, 116, 117, 118, 119 and 121 mass/charge ratio) in accordance with the manufacturer's protocol (AB SCIEX, Framingham, MA, USA). Labeled peptides were pooled and fractionated by strong cation exchange to four fractions. Each fraction was desalted with Sep-Pak C18 Plus Light Cartridge (Waters Corporation, Milford, MA, USA) and analyzed using nano liquid chromatography coupled with tandem mass spectrometry (LC-MS/MS); nano LC-MS was performed on a TripleTOF 5600 mass spectrometer for MS/MS operated with Analyst TF 1.7 software (AB SCIEX, Dublin, CA, USA) interfaced with Eksigent nano LC system using a ChromXP C18-CL column (AB SCIEX). Protein identification and relative quantification was carried out using the ProteinPilot software program, version 5.0 (AB SCIEX). The functions of the various protein contents were determined by searching the UniProt database using the search algorithm within ProteinPilot program (AB SCIEX). Protein ratios were normalized using the overall median ratio for all peptides in the sample for each separate ratio in every individual experiment. Two independent iTRAQ experiments were carried out to profile and quantitate the proteome, and the three technical replicates were used to determine the cut-off for significant fold-changes. A confidence cut-off of >95% was applied for protein identification, and a >1.2-fold change cut-off for all iTRAQ ratios was selected to classify proteins as up- or downregulated.

### Pathway analyses

The pathway analysis is shown in our previous article [47]. Briefly, the IPA software program (Qiagen) was further employed to determine the functional pathways of the identified genes. The IPA software program contains a database of biological interactions among genes and proteins and was used to calculate the probability of relationships among each of the canonical pathways, the upstream pathways and the identified proteins. The IPA program scans the proteins that are entered by the user in order to identify networks using the Ingenuity Pathway Knowledge Base (IPKB) based on interactions between identified proteins and the known and hypothetical interacting genes stored in the program. The IPA program has been licensed for free use since 2013.

### Cell proliferation with EWS/FLI1 or XBP1 siRNA knockdown

For knockdown proliferation studies regarding EWS/FLI1 and XBP1, 6 × 10^5^ ES cells were plated into 6-well plates on day 1. Transfection was performed the same day with 50 nM of the siRNA reagents described above. The cells were counted at 72 h, and all proliferation experiments were performed in triplicate, with the results averaged.

### Growth inhibition assay

The IRE1α-XBP1 pathway inhibitors used were toyocamycin (Tocris Bioscience, Bristol, UK), 2-hydroxy-1-naphthaldehyde (HNA; Sigma-Aldrich), STF-083010 (Sigma-Aldrich), and 3-ethoxy-5 6-dilbromosalicylaldehyde (3ETH; Sigma-Aldrich). ES cells were seeded into 96-well plates at 5000 to 10000 cells/well. The next day, different concentrations of inhibitors or DMSO (as a vehicle control) were added to each well. After 72 h, the inhibitory effect of these inhibitors on the growth of ES cell lines was assessed using a Cell Counting Kit-8 (Dojindo JAPAN, Tokyo Japan) and a Microplate Reader (SAFIRE, TECAN, Männedorf, Switzerland). The IC50 was calculated using the GraphPad Prism program (GraphPad Software, Inc., CA USA).

### Apoptosis (Caspase 3/7) and cell proliferation assays

ES cells were plated into 96-well plates at 5000 to 10000 cells/well, and the next day, inhibitors or DMSO (as a vehicle control) were added to each well. After 24 h, apoptosis (Caspase 3/7 activities) was measured using the Apo-ONE Homogeneous Caspase-3/7 Assay kit (no. G7791; Promega, Madison, WI, USA). The cell proliferation assays were performed as described above.

### *In vivo* animal models

All animal studies were conducted in accordance with the protocols approved by the Animal Ethics Committee of Juntendo University. Prior to injection, ES cells (2.0 × 10^6^) were mixed in PBS with matrigel (BD Biosciences) at a 1:1 ratio. The cell suspension was injected subcutaneously (200 μL/mouse) into the back of 8-week-old female BALB/c nude mice (CREA JAPAN, Tokyo, Japan). When tumors reached approximately 100 to 150 mm^3^ in size, mice were randomized into three groups: intraperitoneal injections of 1.0 or 2.0 mg/kg toyocamycin once a week, or 10% DMSO once a week as a vehicle control. All treatments were administered for 2 weeks (1 day/week). Tumor volumes was measured 3 days a week. The mice were sacrificed after 2 weeks of treatment. Solid tumors on the mice were resected in the end.

### Immunohistochemistry

All tissues were fixed in 10% buffered formalin, embedded in paraffin after routine processing, sectioning, and staining with hematoxylin and eosin (H&E). Immunostaining was performed with antibodies directed to cleaved caspase-3 (#9661, Cell Signaling Technolgy, Danvers, MA, USA). Anti-goat rabbit antibody conjugated to alkaline phosphatase labelled polymer (Nichirei Biosciences INC, Tokyo, Japan) was used to detect antibody binding. Fast red was used as the chromogen.

### Statistical analyses

The relationships between the protein expression, cell proliferations and other factors were analyzed using Fisher's exact test or the *t*-test.

## SUPPLEMENTARY MATERIALS FIGURES AND TABLES


















