# Supplementary material for: IRE1α-XBP1 inhibitors exerted anti-tumor activities in Ewing’s sarcoma
Source: Oncotarget. 2018 Feb 12;9(18):14428–43. doi: 10.18632/oncotarget.24467 (PMC5865680; doi:10.18632/oncotarget.24467)
Supplement: Supplementary file 1 [file oncotarget-09-14428-s001.pdf]

IRE1α-XBP1 inhibitors exerted anti-tumor activities in Ewing's sarcoma

SUPPLEMENTARY MATERIALS

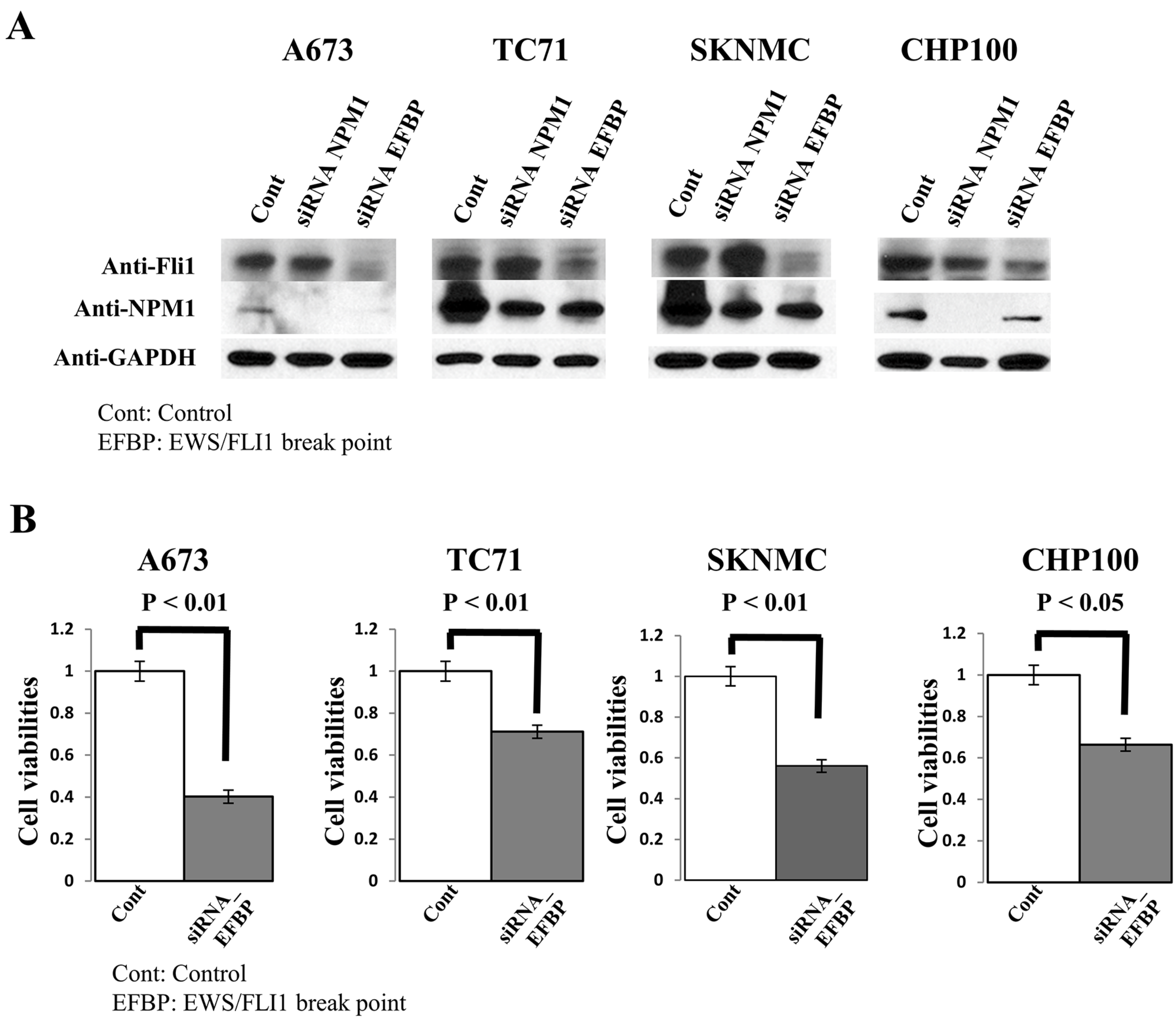

Supplementary Figure 1: EWS/FLI1 knockdown in ES cell lines. (A) The protein expression of FLI1 and NPM1 was verified by Western blotting. Western blotting of FLI1 showed that the cell lines with EWS/FLI1 siRNA knockdown had lower expression of EWS/FLI1 than control cells. Regarding NPM1, our proteomic analyses identified NPM1 as a protein regulated by EWS/FLI1 knockdown. Western blotting using anti-NPM1 demonstrated the suppressed expression of NPM1 in both of the cell lines with either EWS/FLI1 or NPM1 siRNA knockdown. These results confirmed the accuracy of our protein expression profiles by a proteomics approach. **(B)** Regarding the viabilities of the ES cell lines with EWS/FLI1 siRNA knockdown, silencing EWS/FLI1 inhibited the viabilities in all ES cells.

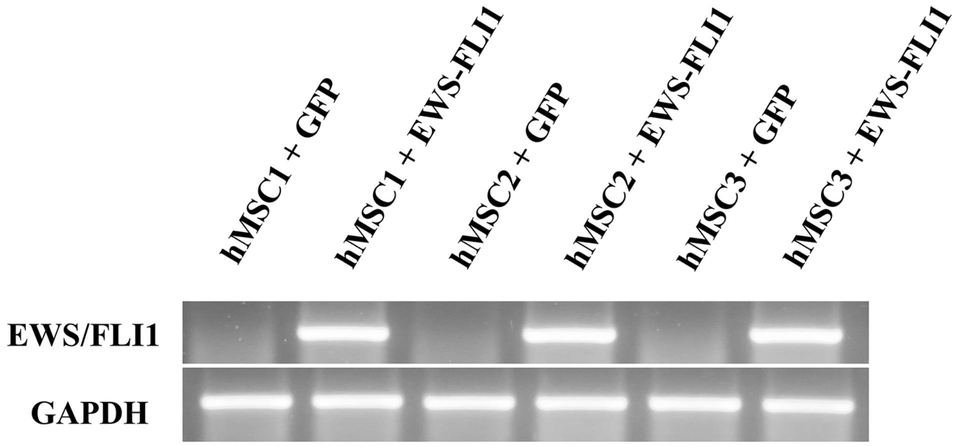

Supplementary Figure 2: Expression of EWS/FLI1 in human mesenchymal stem cell (hMSC) lines. We conducted proteomic studies using expression of EWS/FLI1 in hMSC lines. RT-PCR showed that hMSCs transfected with EWS/FLI1 expressed EWS/FLI1 in all three cell lines.

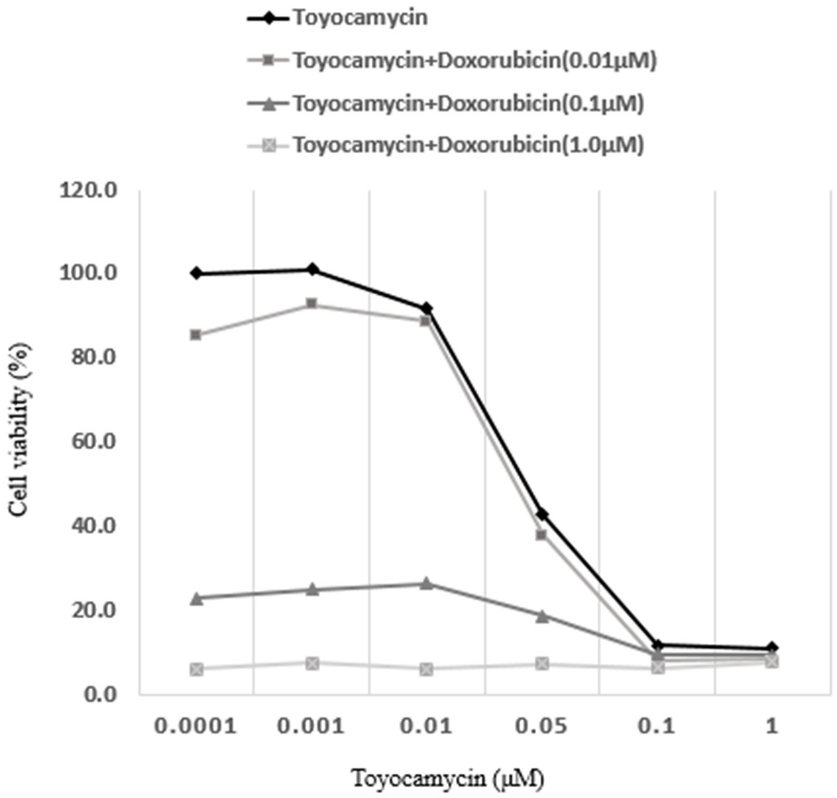

	Toyocamycin (µM)					
	0.0001	0.001	0.01	0.05	0.1	1
Toyocamycin	100.0	100.9	91.7	42.8	11.8	11.0
Toyocamycin+Doxorubicin(0.01µM)	85.5	92.6	88.6	37.8	8.1	8.4
Toyocamycin+Doxorubicin(0.1µM)	22.9	25.0	26.5	18.7	9.7	9.5
Toyocamycin+Doxorubicin(1.0µM)	6.2	7.5	6.1	7.4	6.5	7.8

Supplementary Figure 3. Combination assays (therapies) using doxorubicin and toyocamycin in ES cell lines. In the materials and methods, ES (A673) cells were seeded on 96-well plates (10000 cells/well) and were allowed to adhere overnight. The next day, different concentrations of drugs (single agent: toyocamycin or double agents: toyocamycin and doxorubicin) were added to each well, and the cells were incubated for another 72 h. After incubation, the reagents of a Cell Counting Kit-8 (Dojindo Laboratories, Kumamoto, Japan) were added to each well. After 2 h of incubation, the cellular proliferation was assessed by measuring the absorbance at 450 nm with a microplate reader (SAFIRE, TECAN, Manndorf, Switzerland). In the results, the combination therapies successfully inhibited cell viability in a dose-dependent manner.

Supplementary Table 1: Protein profiles of EWS/FLI1 knockdown in A673.

See Supplementary File 1

Supplementary Table 2: Protein profiles of EWS/FLI1 knockdown in TC71.

See Supplementary File 2

Supplementary Table 3: Protein profiles of EWS/FLI1 knockdown in SKNMC.

See Supplementary File 3

Supplementary Table 4: Protein profiles of EWS/FLI1 knockdown in CHP100.

See Supplementary File 4

Supplementary Table 5: Protein profiles of EWS/FLI1 overexpression in hMSC1 (blue 13 vs. 16).

See Supplementary File 5

Supplementary Table 6: Protein profiles of EWS/FLI1 overexpression in hMSC2 (red 29 vs. 32).

See Supplementary File 6

Supplementary Table 7: Protein profiles of EWS/FLI1 overexpression in hMSC3 (green 37 vs. 40).

See Supplementary File 7

Supplementary Table 8: Protein profiles of EWS/FLI1 overexpression in 3 hMSCs.

See Supplementary File 8

Supplementary Table 9: Surgical materials information

Case	Sex	Age	EWS/FLi1	Type	Body region	XBP1s
Sample 1	Male	48	exon7-exon5	2	Inguinal	+
Sample 2	Male	1	exon7-exon6	1	Peritoneum	+
Sample 3	Male	33	exon7-exon6	1	Thigh	-
Sample 4	Female	61	exon7-exon6	1	Liver/Retroperitoneal	-
Sample 5	Male	1	exon7-exon6	1	Perianal	+
